# Factors influencing the satisfaction level of college students in China: Literature analysis based on grounded theory

**DOI:** 10.3389/fpsyg.2022.1023420

**Published:** 2023-01-24

**Authors:** Qiongying Gu, Guodong Lu

**Affiliations:** ^1^College of Education, Zhejiang University, Hangzhou, Zhejiang, China; ^2^Office of Academic Affairs, NingboTech University, Ningbo, Zhejiang, China

**Keywords:** college students, satisfaction level, influencing factors, qualitative analysis, NVivo, grounded theory

## Abstract

Student satisfaction is an important index for evaluating the quality of higher education and the competitiveness of colleges in China, and most of the current studies on the factors influencing the satisfaction level of colleges students adopt quantitative research methods. A qualitative analysis of 48 literatures on college students’ satisfaction was conducted using NVivo12 in this study. We found that the influencing factors of college students’ satisfaction in China are composed of seven dimensions: school reputation, school environment, personal improvement, organizational management, logistical support, teaching quality, and charges and subsidies. Among them, teaching quality, school environment, organizational management, and logistical support are the core categories. Furthermore, school reputation, school environment, organizational management, logistical support, teaching quality, and charges and subsidies are external factors, while personal improvement is an internal factor. To improve the satisfaction level of college students, the two dimensions of external and internal factors must be taken into account, with external factors being given more attention. This study not only expands the scope of scenarios to which the grounded theory has been applied, but also provides a reference for other scholars to conduct more in-depth empirical studies on college students’ satisfaction in Chinese colleges.

## 1. Introduction

After decades of development, China has established the world’s largest higher education system in terms of the number of students enrolled, and since 2019, Chinese higher education has begun to move into the universalization stage. The gross enrollment rate in higher education reached 57.8% in 2021, with a total enrollment of 44.3 million students, ranking first in the world. Having achieved the goal of giving more people access to higher education, the Chinese people expect high-quality higher education (Li, 2020). A government-led and planned development is a major feature of the Chinese higher education system, which causes complexity in the governance system as Chinese higher education becomes more popular (Hu, 2021). To achieve connotative development and comprehensively improve the quality of higher education, it is necessary to adhere to the basic purpose of “student-centered and student-serving” education (Zhang, 2019, 2020). The Chinese government has proposed a plan to “operate a quality education system that is satisfactory to the people” that aims to give people a sense of reward regarding education development and reform (Wu, 2018), and university students are the main target and the most direct beneficiaries of this system as well as the main target for the quality of higher education evaluation (Wang et al., 2021). Therefore, students’ satisfaction has become an important index for evaluating the quality of higher education and the competitiveness of universities. However, how effectively to determine college students’ satisfaction level is an urgent problem in Chinese higher education at this stage.

Referring to the theory of customer satisfaction, American scholars first conducted theoretical research on college students’ satisfaction in the United States in the 1950s and creatively established it as a concept. Scholars’ analysis of the concept can be divided into two categories. First, satisfaction is the emotional experience of college students, and it refers to the attraction, pride, or positive feelings of college students toward their colleges and universities (Hatcher et al., 1992; Danielson, 1998). This is closely related to their enthusiasm and mental health (Yang et al., 2003) and is an objective evaluation of their colleges and universities according to their internal standards (Lin and Li, 2006). Second, the satisfaction of college students is the result of comparing student expectations and needs. The satisfaction of students depends on the comparison between the expectation of the education demand subject on higher education services and its actual perceived level of education services. It is related to the process of education services and even more so to their effect (Li, 2007), which is a psychological experience (Wei, 2010). Integrating these two perspectives, this study concludes that college students’ satisfaction refers to the degree of positive feelings formed by college students as consumers of college education services as well as their evaluation of college education services and their own educational experiences, educational achievements, and realization of educational expectations. The satisfaction of college students is both an indicator of college students’ learning experience and quality of life and an important criterion for assessing the quality of college education services.

The satisfaction felt by college students is affected by various factors. The United States was the first country to use the college students’ satisfaction scale. Among many student satisfaction scales (SSI), the most widely used ones are the SSI developed by the Noel–Levitz company, the College Students’ Experience Questionnaire (CSEQ) developed by the Center for Postsecondary Research and Planning at Indiana University, the Student Expectation Measurement Scale (cSXQ), the Comprehensive Alumni Assessment Survey (cAAS) of American Higher Education Management System Center, and the Freshman Questionnaire (Ess) used by the Institute of Higher Education in the UK. The Institute of Higher Education and Ipsos MORI jointly designed the College Students’ Satisfaction Scale, which mainly investigates the course learning of new college students in the UK.

In the 1990s, Chinese scholars proposed the concept of satisfaction based on the research of foreign scholars and applied it to the field of higher education. According to the knowledge mapping analysis of research hotspots on college students’ satisfaction levels in Chinese universities, factors that influence satisfaction are a very important research topic (Liu, 2018), and this topic has become prevalent in the field. However, there are still some unaddressed issues that create a gap that requires further study.

First, previous scholars did not develop a college students satisfaction scale that is acceptable to all. In the past two decades, several Chinese scholars conducted research on the influencing factors of Chinese college students’ satisfaction levels either by adopting or adapting foreign model scales. Although these scales comprise various influencing factors and have expressed the level of college students’ satisfaction level from different perspectives, they yielded different results. Second, previous studies on influencing factors of college students’ satisfaction in colleges and universities adopted only quantitative research methods. Thus, it is necessary to use the research results of these previous studies conducted in the past two decades to summarize the empirical generalization from which the composition structure of the influencing factors of college students’ satisfaction level can be understood. Therefore, the objectives of this study are to outline the grounded theoretical thinking framework and research process to be applied to the study; to identity, generalize, and refine the factors influencing college students’ satisfaction levels in Chinese colleges and universities from the existing studies to improve the generality of the results; to attempt to explain the interactions and relationships among the dimensions; and to provide colleges and universities with strategies on how to improve student satisfaction.

The originality of this study lies in the research method used. Moreover, to the best of our knowledge, no scholar has summarized and generalized the results with potential commonality and structure regarding the factors influencing the satisfaction of Chinese college students through a qualitative research method. To achieve our research objectives, we conducted a systematic literature review to identify previously published scientific studies that directly or indirectly focused on this topic.

In summary, based on the grounded theory, this study used a bottom-up qualitative research approach to comprehensively search existing literature on factors influencing Chinese college students’ satisfaction and used NVivo12 qualitative analysis assistance software to filter, summarize, and generalize the literature information to refine the factors that influence Chinese college students’ satisfaction and the dimensional structure to determine the role and interrelationship of each dimension.

## 2. Theoretical framework

**FIGURE 1:**
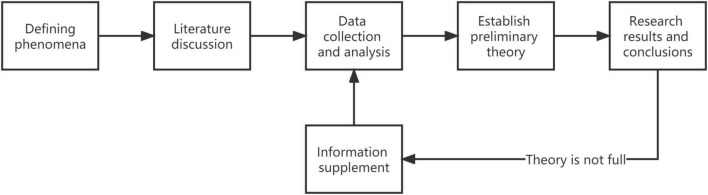
Flow model of grounded theory research.

Grounded theory research is a qualitative research method based on empirical materials that were co-proposed by Glaser and Strauss and start directly from primary sources and abstract theories from the bottom to the top (Wu and Li, 2020). This method advocates not setting something up in advance but collecting data with research questions and finding core concepts and then formulating theories by establishing connections between concepts (Wang and Xu, 2022). It has been pointed out that the grounded theory approach applies to the factor identification category, the interpretation process category, and the analysis of categories that are not easily grasped and exploratory questions on new topics (Jia, 2015). The use of qualitative research methods, especially the grounded theory approach, has become increasingly common in academia. The basic logical thinking structure that researchers mostly tend to use the research process as shown in Figure 1 (Guo, 2015).

**FIGURE 2:**
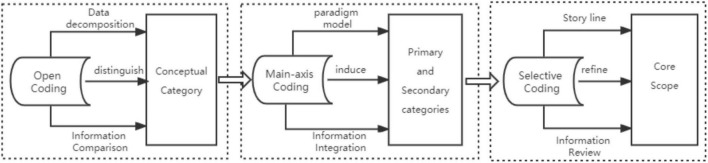
The framework of grounded theory research thinking.

Due to the controversy of the grounded theory method itself and the inconsistency in the use of related concepts and technical tools, the specific application of the method is inconsistent and presents different characteristics. The first one is to start with data collection directly, followed by data coding and refinement and data analysis to form a grounded theory study. The dominant feature of this method is that the data coding adopts an aggregated approach, that is, the open coding, spindle coding, and selective coding commonly used in academia are gathered in the same form, and the coding is completed in an integrated manner and is individually classified as primary coding, focused coding, and another coding (Shan et al., 2011); the second is to omit the analysis of associated literature, personalize the core method data coding into open decoding, spindle decoding, and selective decoding, and complete the conceptualization and category analysis in open decoding (Bai and Liu, 2014); the third is to highlight the interview question design and interviewee information, focus on category categorization of main axis coding and core categories, and form a paradigmatic model of the main category (Wang and He, 2011). There are many other thematic grounded theory research articles, and although the methods used are different, it can be seen that they all follow the thinking framework of grounded theory as shown in Figure 2 (Guo, 2015). The second method, the omission of associated literature analysis, is the one that was used in this study.

## 3. Materials and methods

### 3.1. Study design

This study strictly follows the framework of grounded theory research thinking and abstracts the theory from the bottom to the top level through literature analysis using the following steps: (1) research covering the topic of factors influencing college students’ satisfaction in Chinese universities was selected as the material source of the study; (2) the selected studies were imported into NVivo12 software, and the core data were open-coded to form tertiary nodes, which are the conceptual categories of factors influencing college students’ satisfaction in Chinese universities; (3) the tertiary nodes were generalized to form secondary nodes, which are the secondary categories of factors influencing such satisfaction; (4) the secondary nodes were summarized to form the primary nodes, which are the primary categories of factors influencing this type of satisfaction; and (5) after completing the main-axis coding, selective coding was conducted to determine the core categories of the factors influencing this satisfaction. By constructing the theory through the above analysis, the core influencing factors of satisfaction of Chinese college students and the structural relationship between each factor could be presented in a systematic and orderly way.

### 3.2. Data collection

To obtain original data on the influencing factors of college students’ satisfaction in China, in this study, literature related to the research topic from 2000 to 2021 was selected from the databases of the China Knowledge Network (CNKI), Wanfang, and Vepsa. The search for the selected literature was conducted between December 2021 and January 2022, and the categories of literature included journal papers, dissertations, conference papers, and books. It should be noted that we included a high-quality master’s thesis in our review because the factors influencing college students’ satisfaction in the thesis were specific and detailed. The selected topics included literature on college students’ satisfaction in China that directly studied the influencing factors, the current situation based on the influencing factors, and literature reviews on the influencing factors of educational service quality in Chinese colleges and universities. A total of 551 documents were retrieved, and those targeting specific groups and specific objects were excluded, from which texts covering specific impact factor dimensions were then screened, and 48 documents were confirmed as the research object. The selected literature covers the basic research results in the field of influencing factors of college students’ satisfaction in China over 20 years, and no new codes were found after comparing these results with the literature added after the cutoff time, indicating that the data of this study reached a high degree of saturation.

## 4. Analysis and results

### 4.1. Data coding analysis and model construction

Based on grounded theory, the data analysis process was divided into three stages: open, main-axis, and selective coding. The coding process was assisted by NVivo12. Through the bottom-up induction process, nodes at all levels with subordinate relationships with each other were finally completed.

#### 4.1.1. Open coding: Initial category

Open coding is the basic code-by-code stage, and it aimed to identify phenomena, define concepts, and refine categories (Cui and Luo, 2022). According to the “localization” principle of grounded theory, the naming of open coding concept categories was completely based on the original text in the literature (Zhou and Li, 2019). By combining, confirming, and coding the contents of the 48 documents in the sample, 181 tertiary nodes were obtained from 284 reference points. The tertiary nodes, which are located at the bottom layer of the subordinate relationship, are the initial category of factors influencing college students’ satisfaction in Chinese universities.

#### 4.1.2. Main-axis coding: Primary and secondary categories

Main-axis coding is based on open coding, combining related theories, regrouping concepts, and discovering and establishing organic connections among conceptual categories, that is, categories to express the logical relationships among the parts of the original material. First, the initial concepts formed in the open coding stage are examined, clustered, and analyzed, and subcategories are defined and assigned; second, the subcategories are linked and summarized according to certain logical structural relationships to form the main categories; again, the main category set is constructed; finally, a structural model based on the category relationships is built (Cui and Luo, 2022).

**FIGURE 3:**
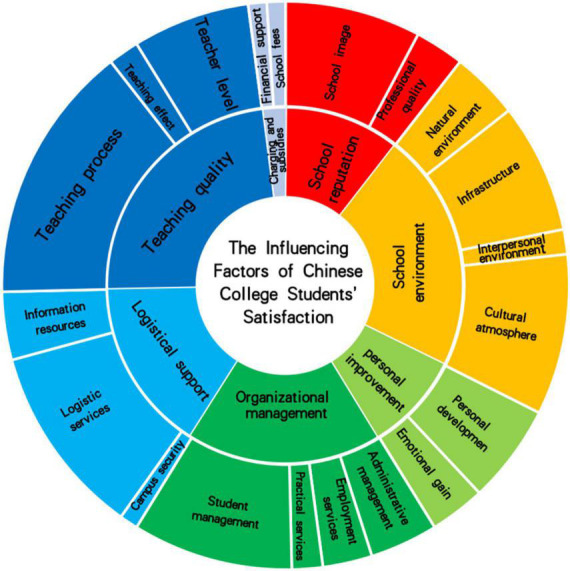
Structural model of influencing factors of college students’ satisfaction. Due to the large number of tertiary nodes, only primary and secondary nodes are listed in the figure.

Using NVivo12 software, 181 tertiary nodes were summarized into 20 secondary nodes, which were located at the middle layer of the subordinate relationship. The secondary nodes were formed through further induction and integration of the tertiary nodes, which are the sub-categories of the factors influencing the satisfaction of college students in Chinese universities. The 18 secondary nodes were further summarized and integrated, and seven primary nodes were obtained, namely: school reputation, school environment, organizational management, teaching quality, logistical support, charges and subsidies, and personal improvement. The primary nodes, which are the main category of factors influencing college students’ satisfaction in Chinese universities, were located at the top layer of the subordinate relationship refer to Table 1 for details. Figure 3 shows the structural model of the influencing factors of college students’ satisfaction in China.

**TABLE 1:** Coding statistics of factors influencing satisfaction of Chinese university students.

Primary nodes	Secondary nodes	Tertiary nodes (181)
Teaching quality (66)	Teaching process (42)	Instructional services (13), instructional management (5), instructional process (2), instructional course management (2), curriculum and instruction (2)……
	Teaching effect (5)	Ability to analyze problems (1), teaching feedback (1), teaching perception (1), teaching effectiveness (1), and teaching quality (1).
	Teacher level (19)	Teacher teaching (5), faculty (3), teacher teaching attitude (1), teacher personal level (1), teacher professionalism (1)……
School environment (62)	Natural environment (11)	Life and environment (3), campus environment (2), natural environment (2), environment (1), school environment (1)……
	Infrastructure (21)	Learning conditions (4), teaching conditions (2), teaching and support facilities (1), hardware facilities (1), living facilities (1)
	Interpersonal environment (4)	Interpersonal relationships (3), interpersonal environment (1)
	Cultural atmosphere (26)	Campus culture (5), campus life (4), campus climate (3), human environment (2), campus cultural climate (2)……
Organizational management (49)	Administrative management (10)	Consulting and advising (2), ability to handle matters (1), management services (1), management and support services (1), administrative support (1)……
	Employment services (8)	Career services (2), career guidance (2), admissions and employment (2), employment (1), career guidance and assistance (1)
	Practice services (5)	Social practice (1), club activities (1), practical innovation (1), practical skills training (1)
	Student management (26)	Student administration (4), student work (3), student administration and support (3), student administration and support services (2), student support and services (2)……
Logistical support (46)	Campus security (4)	On-campus safety (1), campus security (1), security situation (1), security environment (1)
	Logistic services (31)	Logistics services (12), life services (3), logistical support (2), canteen (2), accommodation management (2)……
	Information resources (11)	Library (4), library services (3), information resources (2), network status (1), network resources (1).
School reputation (30)	School image (22)	Image of the school (12), image of the university (1), image of the brand (1), image of the college (1), image of the university campus (1)
	Professional quality (8)	Professional development (2), professional programs (1), professional curriculum (1), professional interests (1), professional quality (1)……
Fees and subsidies (6)	School fees (3)	Fees (1), price of fees (1), school fees (1)
	Financial support (3)	Financial support (1), financial support (1), funding (1).
Personal improvement (25)	Personal development (16)	Self -development (4), personal development (4), student development (1), self enhancement and development (1), cognitive and competency gain (1)……
	Emotional gain (9)	Emotion (2), care for the individual (1), reliability (1), personal gain (1), effect (1)……

The number in parentheses is the number of reference points.

The model presents a ring structure with the center spread around it. The center is the theme of the model, that is, the influencing factors of college students’ satisfaction. The multi-layer ring is the specific hierarchical structure of the influencing factors of college students’ satisfaction: The internal node division of each ring layer reflects the dimensional category of the influencing factors. According to the model, the influencing factors of college students’ satisfaction in China are composed of seven primary nodes and 20 secondary nodes. The vertical structure of each sector of the ring reflects the hierarchical relationship of the influencing factors of satisfaction. The area of each sector in the ring is related to the number of coding reference points, which represents the number of documents supporting a node that reflects an influencing factor. Among the primary nodes, the teaching quality node has the largest number of reference points and the most important impact. The school environment, organizational management, and logistical support also have a large number of reference points and play an important role in the whole model. School reputation and personal improvement have a relatively small number of reference points, which has a certain impact on the whole. Charges and subsidies have the least number of reference points and have a weak impact. In all secondary nodes, the teaching process received the most reference point support, suggesting that this basic demand is still an important factor affecting the satisfaction of college students. Logistics service, cultural atmosphere, student management, school image, infrastructure, and teacher level have more reference points and have attracted more literature attention. These nodes are also very influencing factors of college students’ satisfaction.

#### 4.1.3. Selective coding: Core categories

Selective coding is based on spindle coding to identify core categories and establish the relationship between core categories and other categories (Cui and Luo, 2022). By abstracting the seven nodes from the axial coding at a higher level, the factors affecting the satisfaction of Chinese college students can be divided into two dimensions: external and internal factors. External factors are defined as external objective factors not controlled by the students themselves. These factors have great management flexibility and do not differ due to the individual differences of students (Zhou and Li, 2019). The impact of these factors on college students’ satisfaction can be adjusted through management means, which is the main direction for improving college students’ satisfaction in the future. Internal factors are defined as students’ sense of self-acquisition, which is greatly affected by their individual subjective tendencies and differences, and external intervention is relatively ineffective on these factors (Zhou and Li, 2019). To sum up, school reputation, school environment, organizational management, logistical support, teaching quality, and charges and subsidies are classified as external factors, while personal improvement is classified as an internal factor. The reference points of the external factor code account for 91.2% of the whole, which identifies the external factors as the main source of the core category. The reference point of the internal factor code accounts for 8.8%, so the internal factors can be classified as secondary factors.

External factors include four primary nodes (teaching quality, school environment, organizational management, and logistical support) that have a direct impact on the school reputation node. From the point of view of coding reference points, these five nodes account for 81.4% of all nodes and have global commands. Further considering the role of nodes in the whole and removing the charges and subsidies (2%) and personal improvement nodes (8%), which account for the smallest proportions, teaching quality, school environment, organizational management, and logistical support can be determined as the core categories of the influencing factors on Chinese college students’ satisfaction.

### 4.2. Model interpretation and relationship analysis

This study focuses on the four high-frequency nodes of the core categories of the model: teaching quality, school environment, organizational management, and logistical support; the two categories of fees and funding and personal enhancement are low-frequency nodes, and few researchers focused on this perspective, so they will not be explained in depth.

#### 4.2.1. Teaching quality as a factor of direct influence

Teaching quality refers to the teaching and learning process and the effects it produces as well as the extent to which the established teaching and learning objectives are met (Wu, 2019). The teaching quality node has the most reference points of all primary nodes. It is the most direct influencing factor affecting college students’ satisfaction. According to the number of coded reference points, the teaching quality node includes three secondary nodes: teaching process, teacher level, and teaching effect, with a total of 66 reference points, accounting for 23.3% of the overall number of reference points. Among the three secondary nodes included in teaching quality, the teaching process node contains 42 reference points, which is the largest number. The teaching process is related to the acquisition of students’ knowledge, abilities, and qualities (Peng and Liu, 2022). From the included tertiary nodes, it can be seen that students not only pay attention to teaching content and methods but also to extracurricular counseling. Therefore, improving the quality of the teaching process is an important means to improving college students’ satisfaction.

The teacher-level node contains 19 reference points. The teacher level is the cornerstone of the quality of the teaching process (Luo et al., 2019), and the implementation of the teaching link depends on teachers. Students care not only about teachers’ teaching and academic levels but also their attitude. The teaching effect includes five reference points that reflect the students’ feedback on teaching quality and is a test of the teaching process (Peng and Liu, 2022). Table 2 shows the nodes at all levels and coding reference points included in the teaching quality node.

**TABLE 2:** Nodes and coding reference points in teaching quality.

Secondary node	Level 3 node	Number of reference points encoded	Secondary node	Level 3 node	Number of reference points encoded
Teaching process (42)	Teaching method	1	Teaching effect (5)	Teaching results	1
	Teaching services	13		Teaching feedback	1
	Teaching guidance	1		Teaching perception	1
	Teaching management	5		Teaching efficiency	1
	Teaching process	2		Teaching quality	1
	Teaching course	1	Teacher level (19)	Staff team	1
	Teaching course management	2		Teacher	1
	Content of courses	1		Teacher service	1
	Teaching materials	1		Teacher’s personal level	1
	Educational resources	1		Teacher teaching	5
	Curriculum and teaching	2		Teacher teaching attitude	1
	Curriculum teaching	1		Teacher and teaching	1
	Course teaching and other management	1		Professional quality of teachers	1
	Course content	1		Teaching academic	1
	Classroom teaching	1		teachers	1
	Extracurricular teaching	1		Teaching staff	3
	Master theoretical knowledge	1		Faculty	1
	Teacher-student interaction	2		Academic factors	1
	Teaching method	1			
	Outreach learning	1			
	School teaching level and teaching environment	1			
	Academic support	1			

The numbers in parenthesis are the sum of the own encoding reference points and the tertiary nodes’ encoded reference points. The same applies to the tables that follow.

#### 4.2.2. School environment as an important influencing factor

School environment refers to the physical environment, hardware facilities, and humanistic state of the school (Zhang, 2018), and it is an important factor affecting the satisfaction of college students. It includes four secondary nodes: cultural atmosphere, infrastructure, natural environment, and interpersonal environment, with a total of 62 reference points (21.9%). Among the four secondary nodes, the cultural atmosphere node contains 26 reference points at most. The cultural atmosphere reflects the overall humanistic environment of the school (Xiao and Jing, 2012). From the tertiary nodes of cultural atmosphere, it can be seen that students are not only concerned about the overall atmosphere of the school but also about its cultural construction and atmosphere construction. The infrastructure node contains 21 reference points. It is a relatively important node that mainly refers to the school’s hardware facilities for teaching, teaching assistance, and school life. The natural environment node contains 11 reference points. It mainly pertains to the physical environment of the campus. The interpersonal environment includes four reference points and mainly refers to the interpersonal environment of college students. Among the four nodes of the school environment, the cultural atmosphere and interpersonal relationship nodes represent humanistic factors, which can be further summarized as humanistic condition nodes, with a total of 30 reference points. The natural environment and infrastructure nodes represent basic conditions, which can be further summarized as basic condition nodes, with a total of 32 nodes. This indicates that the construction of humanistic conditions and basic conditions are equally important in improving students’ satisfaction levels. Table 3 shows the nodes at all levels and coding reference points included in the school environment node.

**TABLE 3:** Nodes and coding reference points in school environment.

Secondary node	Level 3 node	Number of reference points encoded	Secondary node	Level 3 node	Number of reference points encoded
Infrastructure (21)	Service facility	1	Cultural atmosphere (26)	Cultural environment	2
	Infrastructure	1		Cultural context	1
	Basic conditions	1		Cultural construction	1
	Teaching facilities	1		Recreational and sports activities	1
	Teaching facilities	1		Entertainment and safety	1
	Teaching infrastructure	1		Entertainment and sports	2
	Teaching facilities	1		School spirit	1
	Teaching facilities and equipment	1		Campus atmosphere	3
	Teaching conditions	2		Campus ethos	1
	Teaching conditions and utilization	1		School life	4
	Facility and equipment	1		Campus culture	5
	Domestic installation	1		Campus cultural atmosphere	2
	Campus facilities	1		School environment	2
	Learning conditions	4	Natural environment (11)	Environment	1
	School construction	1		Life and environment	3
	Hardware facilities	1		Campus environment	2
	Self-study environment	1		Campus environment and public security	1
Interpersonal environment (4)	Interpersonal relation	3		School environment	1
	Interpersonal environment	1		Hardware environment	1
				Natural environment	2

#### 4.2.3. Organizational management as a factor of indirect influence

Organizational management in this context refers to the management activities carried out by the institution based on the establishment of an organizational structure and clear relationships of authority (Huang, 2019). Organizational management is an indirect factor affecting satisfaction levels. The organizational management node, with a total of 49 reference points, includes four secondary nodes: student management, administrative management, employment services, and practical services. Student management occupies an important position with the highest number of reference points at 26. Administrative services include 10 reference points, mainly management system, management service quality, and consulting guarantee. Employment service includes eight reference points that refer to the employment guidance and assistance obtained by students. Practical services include five reference points. The number of reference points of each tertiary node and the gap between them is small, indicating that little attention is given to practical service as an influencing factor in the existing literature. The impact of this factor on overall satisfaction has been ignored or needs to be further studied. We believe that many factors contained in organizational management serve the work of the teaching center of the school and have an indirect impact on satisfaction. Table 4 shows the nodes at all levels and coding reference points included in the organizational management node.

**TABLE 4:** Nodes and coding reference points in organizational management.

Secondary node	Level 3 node	Number of reference points encoded	Secondary node	Level 3 node	Number of reference points encoded
Student management (26)	Assistant	1	Administrative management (10)	Ability to handle affairs	1
	Counselor quality	1		University management	1
	Counselors’ support	1		Management service	1
	Management of students	1		Management and support services	1
	Student development management	1		Management system	1
	Student work	3		Administrative security	1
	Student attention	1		Consultancy and advice	2
	Student management	4		Resources and consulting	1
	Student management	1		Integrated management	1
	Student management mechanism	1	Employment services (8)	Obtain employment	1
	Student management and support	3		Employment service	2
	Student management and support services	2		Employment guidance	2
	Daily living conditions of students	1		Employment guidance and help	1
	Student life	1		Admission and employment	2
	Students’ needs	1	Practice services (5)	Social practice	1
	Student support and service	2		Social practice	1
				Club events	1
	Student support and management services	1		Practice innovation	1
				Practice ability training	1

#### 4.2.4. Logistical support as a controllable impact factor

Logistical support is an important prerequisite and guarantees universities to carry out education and teaching, including both service and administrative content (Wang, 2021). Logistical support is a controllable influencing factor of college students’ satisfaction in China. The logistics support node has a total of 46 reference points and includes three secondary nodes: logistics services, information resources, and campus security. It has a total of 31 reference points, which is the highest number among all 20 secondary nodes, indicating that students are highly concerned about the quality of the logistics services of the university. Improving the quality of logistics services can effectively improve the satisfaction of college students to a certain extent. Information resources include 11 reference points, mainly library and network resources. Campus security includes four reference points. The number of reference points of each tertiary node, the gap between them, and its impact are small. Many factors contained in the logistical support node can be changed indirectly through the factors contained in the campus environment and organizational management node to establish an association between the support between the nodes and achieve indirect control over the logistical support factors. Table 5 shows the nodes at all levels and coding reference points included in the logistical support node.

**TABLE 5:** Nodes and coding reference points in logistical support.

Secondary node	Level 3 node	Number of reference points encoded	Secondary node	Level 3 node	Number of reference points encoded
Logistic services (31)	Restaurant service	1	Information resources (11)	Library	4
	Service and guarantee	1		Library services	3
	logistics	1		Network conditions	1
	Logistics safety	1		Net resource	1
	Logistical support	2			
	Logistics service	12		Information resources	2
	Logistics management and Services	1			
	Logistics life service	1	Campus security (4)	Campus safety	1
	Service for life	3			
	Dining room	2		Campus security	1
	Accommodation conditions	1			
	School infirmary	1		Public security situation	1
	Dorm	1			
	Hospital service	1		Public security environment	1
	Accommodation management	2			

### 4.3. Results

#### 4.3.1. Satisfaction-influencing factors present a circular structure model

The influencing factors on college students’ satisfaction level in China are composed of a three-layer ring structure model. The first layer is the macro influencing factors of college students’ satisfaction, and it includes seven primary nodes. Among the nodes, teaching quality, logistical support, and school environment contain the most reference points. The second layer is the intermediate influencing factors. It includes 20 secondary nodes, of which the teaching process, logistics services, student management, and cultural atmosphere contain the most reference points. The third layer is the micro influencing factors, and it includes 181 tertiary nodes, of which teaching service, logistics services, and school image contain the most reference points. A node containing a high number of reference points can be regarded as a factor that has a greater impact on the satisfaction of college students and key factor schools can work on to improve the satisfaction of college students.

#### 4.3.2. Satisfaction-influencing factors belong to different categories

First, the influencing factors of college students’ satisfaction in China were divided into external and internal factors. School reputation, school environment, organizational management, logistical support, teaching quality, charges, and subsidies were classified as external factors. These factors have great management flexibility and are not different due to the individual differences of students. The impact of these factors on college students’ satisfaction can be adjusted through management means. Personal improvement is an internal factor, which is greatly affected by individual subjective tendencies and differences, and external intervention is relatively ineffective on such factors. External factors dominate the influencing factors of college students’ satisfaction, and they represent the main direction for the school to improve the satisfaction of college students in the future.

Second, teaching quality, school environment, organizational management, and logistical support are the “core categories” of the influencing factors. Charges and subsidies and personal improvement have the least impact and were considered separately as “other categories.” From the analysis of the degree of influence and specific content of college students’ satisfaction, teaching quality is a direct influencing factor, the school environment is an important influencing factor, organizational management is an indirect influencing factor, and logistical support is a controllable influencing factor. The “core category” is an important element that schools can manage to improve the satisfaction of university students.

#### 4.3.3. Relationship between satisfaction-influencing factors

First, we would like to discuss the relationship between dimensions in terms of importance. The number of reference points of influencing factors can indicate the relationship between dimensions in terms of importance, and the number of included reference points can be regarded as the size of the influence of the factor on the satisfaction of college students in Chinese universities, which can determine whether a factor is a key factor for the school to manage to improve the satisfaction of college students.

Second, we would like to discuss the relationship between the mechanism of action among the dimensions. The external and internal characteristics of the factors influencing the satisfaction of college students in Chinese universities have different mechanisms of action. The external factors can be regulated by external interventions, while the internal factors have individual subjective tendencies and are influenced by external factors that have effects on individuals.

Third, we would like to discuss the relationship of influence effect among the dimensions. The effect of “core categories” is more clear than that of “other categories”; the effect of direct factors is more direct than that of indirect factors; the effect of important factors is more significant than that of controllable factors.

## 5. Discussion

Most previous studies on the satisfaction of college students are based on the theoretical foundation of traditional evaluation index systems. They focused on the composition of measurement indicators and only divide one issue into many different levels to study that are relatively independent of each other without studying their correlations and without fundamentally revealing the internal relationship between these variable indicators (Li, 2011). In addition to identifying, generalizing, and distilling the factors influencing university students’ satisfaction in China from existing studies, the authors of this study were more concerned with the interactions and relationships between the dimensions of the influencing factors.

### 5.1. Theoretical implications

This study has several key theoretical contributions. First, based on the literature data, the linkage between the factors influencing college students’ satisfaction in Chinese colleges and universities has been established, and the theory is anchored in the grounded theory approach. Through grounded theory, this study also indicates that the factors influencing college students’ satisfaction in Chinese colleges and universities consist of a three-layer ring structure model: macro-influencing factors of college students’ satisfaction, intermediate influencing factors of college students’ satisfaction impact, and micro-influencing factors of college students’ satisfaction.

Second, this study classifies the factors influencing the satisfaction of college students in China into two categories of external and internal factors through qualitative analysis of the data. Previous studies focused on the specific content of the influencing factors of college students’ satisfaction; however, they did not consider that different types of influencing factors have different mechanisms of action and, therefore, the adjustment mechanism is also different (Liu et al., 2022). This study shows that the external factors do not vary according to students’ individual differences and have large management flexibility. Additionally, the influence of these factors on college students’ satisfaction can be adjusted by management means. In contrast, internal factors are more influenced by students’ individual subjective tendencies and individual differences, and external interventions are relatively ineffective.

Third, this study introduces grounded theory into the study of the factors influencing students’ satisfaction in colleges and universities, which expands the scope of the scenarios to which the theory has been applied and complements previous studies. Unlike previous research, this study used grounded theory to classify the factors influencing the satisfaction level of Chinese college and university students into “core categories” and “other categories” factors. The “core categories” is the most important factor for schools to manage when improving students’ satisfaction level in Chinese colleges and universities.

### 5.2. Practical implications

Our study has practical significance for measuring college students’ satisfaction levels. First, this study has formed the measurement dimensions of college student satisfaction in Chinese colleges and universities as well as the roles and interrelationships among the dimensions, and it can be used as a basis for colleges and universities to develop a scale for measuring college student satisfaction on the one hand; however, it can provide a basis for other scholars to conduct more in-depth empirical research on college student satisfaction in Chinese colleges and universities.

Second, we found that there are differences in the degree of influence (between the “core category” and “other categories”) on satisfaction among various factors and nodes of satisfaction of college students in Chinese universities. Among these factors, teaching quality, school environment, organizational management, and logistics support are the core categories, and they represent key areas that colleges and universities can manage to improve college students’ satisfaction. Targeted and continuous improvement in key areas can significantly improve college students’ satisfaction; however, some factors have “external causes” while others have “internal causes.” School reputation, school environment, organizational management, logistical support, teaching quality, and fees and subsidies are external influencing factors. For these factors, the school can adjust its characteristics by improving the system, increasing investment, communication, and publicity, and engaging in other external interventions to improve the level of student satisfaction.

Third, among all the influencing factors, we found that teaching quality has the highest number of reference points among all the primary nodes, meaning it is not only the most direct influencing factor of college students’ satisfaction but also the most critical factor for improving college students’ satisfaction. Combined with the content of its secondary nodes, we suggest that universities can improve the teaching quality of their schools by strengthening the training of teachers, standardizing the teaching process, enriching teaching resources, improving the quality of classroom teaching, and responding to teaching feedback in a timely manner.

### 5.3. Limitations and future research

Despite its significance, this study still has some limitations. First, only literature related to the factors influencing college students’ satisfaction in Chinese universities was collected for excavation, and the final node content was relatively common, failing to explore “emotional tool support” (Liu and Tao, 2017), “faculty humanistic care” (Min, 2019), and “student identity” (Chen and Fang, 2018), which researchers believe really makes a difference in practice and also influence students’ satisfaction. Therefore, future studies can expand the scope of literature sources and add research methods such as in-depth interviews to further enrich the dimensions of factors influencing college students’ satisfaction and improve the theoretical structure.

Second, the measurement dimensions of college students’ satisfaction formulated in this study based on 48 literature sources that were qualitatively analyzed have not been analyzed empirically to verify the reliability of the measurement dimensions and find correlations. Therefore, future research should be based on the satisfaction measurement dimensions formulated in this study, select samples to implement the measurement practice and verify the reliability and validity of the satisfaction measurement dimensions of college students in Chinese universities through descriptive statistical analysis, and reliability and validity tests of the survey data and the relationships among the dimensions should be further explored through correlation analysis.

Third, the results of the study showed that the dimensions of factors influencing college students’ satisfaction levels were not compared with the classic foreign satisfaction scales or with the dimensions of factors influencing college students’ satisfaction in other countries; therefore, this study did not sufficiently analyze the connotation of Chinese sociocultural conditions in the measurement dimensions. In the future, researchers should further consider the factors influencing the satisfaction of Chinese college students and their differences with other countries in the national context through the method of comparative analysis.

## 6. Conclusion

The results of this study showed that, based on grounded theory, the main dimensions and hierarchical structure of the factors influencing college students’ satisfaction in Chinese universities can be outlined and constructed, and the interactions and relationships of each dimension can be determined. The structural model of factors influencing college students’ satisfaction in Chinese colleges and universities is a three-level ring structure with different reference points; based on the types of factors, they can be divided into external and internal factors; based on the degree of influence, they can be divided into “core categories” and “other categories.” In the management of colleges and universities, to improve the satisfaction level of college students, we should focus on external factors and take into account the two dimensions of external and internal factors, and we should consider the influence of core categories such as teaching quality, school environment, organizational management, and logistical support on the satisfaction level of Chinese college students in colleges and universities.

## Data availability statement

The original contributions presented in this study are included in the article/supplementary material, further inquiries can be directed to the corresponding author.

## Author contributions

QG and GL: conceptualization, formal analysis, data curation, and methodology. QG: writing—original draft preparation and project administration. GL: writing—review and editing. Both authors have read and agreed to the published version of the manuscript.
